# Genotypic and phylogenetic characterization of *Salmonella* Minnesota ST548 strains with potential risk to public health

**DOI:** 10.1007/s42770-026-02071-y

**Published:** 2026-10-23

**Authors:** Camila de Fátima Nascimento, Mauro M S Saraiva, Valdinete Pereira Benevides, Isabella Cardeal Campos, Isis Mari Miyashiro Kolososki, Heitor Leocádio de Souza Rodrigues, Daniel Farias Marinho do Monte, Herrison Fontana, Fernanda Esposito, Nilton Lincopan, Saura Rodrigues da Silva, Vitor Fernandes Oliveira de Miranda, Angelo Berchieri Junior, Oliveiro Caetano de Freitas Neto

**Affiliations:** 1 Postgraduate Program in Veterinary Sciences, School of Agricultural and Veterinary Sciences, São Paulo State University (Unesp), Jaboticabal, 14884-900 Brazil; 2 Department of Pathology, Reproduction and One Health, School of Agricultural and Veterinary Sciences, São Paulo State University (Unesp), Jaboticabal, 14884-900 Brazil; 3 Department of Population Health and Pathobiology, North Carolina State University, College of Veterinary Medicine, Raleigh, NC USA; 4 Department of Clinical Analysis, School of Pharmaceutical Sciences, University of São Paulo, São Paulo, Brazil; 5 Department of Microbiology, Institute of Biomedical Science, University of São Paulo, São Paulo, Brazil; 6 Laboratory of Plant Systematics and Genomics, Department of Biology, School of Agricultural and Veterinary Sciences, São Paulo State University (Unesp), Jaboticabal, 14884- 900 Brazil; 7 CBioClima - Center for Research on Biodiversity Dynamics and Climate Change, São Paulo State University (UNESP), Rio Claro, 13506-900 Brazil; 8 Department of Preventive Veterinary Medicine, School of Veterinary, Federal University of Minas Gerais (UFMG), Belo Horizonte, CEP: 31270-010 Minas Gerais Brazil

**Keywords:** Beta-lactamases, Food epidemiology, Plasmid, Resistance, Surveillance

## Abstract

*Salmonella* Minnesota ST548 has emerged as an important serovar in the poultry industry and has been detected in food products. Five nonclonal *S.* Minnesota isolates from poultry in southeastern Brazil were characterized using whole-genome sequencing (WGS), and 263 database sequences were analyzed for virulence genes, antimicrobial resistance genes, and phylogenetic relationships. Among the analyzed sequences, 191 multidrug-resistant (MDR) strains carried antimicrobial resistance genes (ARGs) conferring resistance to aminoglycosides (*aac(6′)-Iaa*, *aph(3′)-Ia* and *aadA1*), quinolones (*qnrB19*), tetracycline (*tetA*), sulfonamides (*sul2*), and beta-lactams (*bla*_CMY−2_, *bla*_CTX-M-8_, *bla*_CTX−M−55_ and *bla*_TEM−126_). Furthermore, several genes associated with virulence factors were identified, including adherence (*faeC*,* faeE*,* and fimB*), fimbrial adherence determinants (*pegA*,* staA*,* fimA*,* and csgD*), magnesium uptake (*mgtB* and *mgtC*), macrophage inducible genes *(mig-14*), toxin (*cdtB*) and secretion system (*ssaM*,* sseE* and *sseJ*). Twenty-five plasmid replicons and 53 distinct prophages were identified in the analyzed sequences. Phylogenetic analysis showed close relationships between nonclonal *S*. Minnesota poultry isolates from southeastern Brazil and *S*. Minnesota sequences associated with human outbreaks from other sources and countries. These findings highlight the need for surveillance of this serovar in both public and veterinary health.

**Supplementary Information:**

The online version contains supplementary material available at https://doi.org/10.1007/s42770-026-02071-y.

**Impacts**:

- *Salmonella* Minnesota (SM) strains from Brazilian poultry may harbor multiple resistance and virulence determinants, posing a potential risk to public health.
- *Salmon*e*lla* Minnesota strains from poultry carry genes associated with resistance to antimicrobials and disinfectants used in hospital and animal-production environments.
- Mobile genetic elements and prophage regions identified in SM sequences may facilitate the dissemination of resistance and virulence genes and support molecular subtyping of pathogens relevant to One Health.

## Introduction

Nontyphoidal *Salmonella* (NTS) is an enteric pathogen frequently associated with a substantial global health and economic burden [1]. In 2017, an estimated 553,000 invasive cases of *Salmonella* spp. occurred, resulting in 77,500 deaths [2]. Poultry products, particularly broiler meat, are frequently implicated in foodborne outbreaks [2, 3]. Although NTS typically causes self-limiting diarrheal disease in immunocompetent individuals, populations at increased risk, including children, older adults, people with human immunodeficiency virus, and individuals undergoing cancer treatment, may develop invasive disease that can lead to sepsis and death [3, 4].

In major poultry meat-producing countries, including Brazil [5], *Salmonella* Minnesota has emerged as one of the most prevalent serovars in commercial broilers [6], and many isolates exhibit high molecular similarity [7]. Nevertheless, few human cases of *S*. Minnesota infection have been reported, although outbreaks with serious clinical consequences, including two deaths among older adults, have occurred [8, 9]. Therefore, the dissemination of this serovar from the broiler industry and its potential public health impact warrant continued surveillance.

Of particular concern, NTS can infect a broad range of hosts, including domestic animals, wild and farmed birds, and humans, and can carry resistance and virulence determinants whose dissemination among pathogens represents a major One Health challenge [10–12]. Pathogens carrying antimicrobial resistance genes (ARGs) can occur in both hospital and animal-production environments, where antimicrobial agents are used [11, 12]. Moreover, virulence genes and ARGs can disseminate via mobile genetic elements, such as plasmids and transposons, through horizontal gene transfer or bacteriophage-mediated transduction [10].

Despite increasing interest in this serovar, important gaps remain regarding potential risks to public and veterinary health. Therefore, this study aimed to phenotypically and genotypically characterize five nonclonal *Salmonella* Minnesota isolates from poultry in southeastern Brazil and to conduct comparative genomic analyses of ARGs, virulence genes, plasmids, and prophages and to infer phylogenetic relationships among all analyzed genomes.

## Materials and methods

### Sample collection

Five nonclonal isolates of *S.* Minnesota were selected from the collection of TECSA Laboratory located in Belo Horizonte, Minas Gerais, State of Brazil, and obtained from poultry feces during official poultry farms monitoring; samples were collected in the state of São Paulo, southeastern region of Brazil, during 2019 (SM_04) and 2020 (SM_01, SM_02, SM_03 and SM_05) in selective culture media MacConkey (Kasvi, Madrid, Spain) and XLT4 (Xylose Lactose Tergitol 4 - Kasvi, Madrid, Spain). Serotyping confirmation of the samples was carried out at the *Enterobacteriaceae* Laboratory of the Oswaldo Cruz Foundation (Fiocruz).

### Whole-genome sequencing (WGS) and downstream analysis

Genomic DNA of the five nonclonal *Salmonella* Minnesota isolates from poultry in southeastern Brazil was extracted from an overnight culture in LB broth (Kasvi, Madrid, Spain) using the PureLink^®^ Genomic DNA according to the manufacturer’s instructions. DNA quality and concentration were evaluated using Qubit 2.0 fluorometer (Life Technologies, Carlsbad, USA), and genomic libraries were prepared using a Nextera DNA Flex Library Preparation Kit (Illumina Inc., San Diego, CA). Afterwards, DNA sequencing was performed on an Illumina NextSeq550 platform using a paired-end protocol (2 × 75-bp). Short reads were quality checked and trimmed with TrimGalore v0.6.10 (https://github.com/FelixKrueger/TrimGalore), using PHRED33. Subsequently, the genomes of nonclonal *S*. Minnesota isolates from poultry in southeastern Brazil were assembled by *De Novo* using the CLC bioGenomics Workbench version 6.0 program (Aarhus, Denmark). The resulting assembled sequences were automatically annotated with RAST 2.0 [13]. In addition to the five nonclonal *S*. Minnesota isolates from poultry in southeastern Brazil, 263 assembled *S*. Minnesota worldwide sequences, isolates over the past 24 years, were obtained from the Enterobase database [14, 15]. The genomes were downloaded from 17 May 2024 to 22 May 2024 from Enterobase, according to the authors’ standards, using the following filters: *S*. Minnesota, ST548, and coverage ≥ 90, all recovered in fasta format. Further details of each of the isolates are available in the Supplementary Materials (Table S1).

All 268 assembled sequences underwent in silico analysis utilizing bioinformatics tools available from the Center for Genomic Epidemiology (CGE), including the identification of *Salmonella* Pathogenicity Islands (SPIs) (SPIFinder 2.0, accessed on 21 Sep 2024) [16]; presence of ARGs (ResFinder 4.1, 26 Jun to 23 Jul 2024) [17]; detection of plasmid replicons (PlasmidFinder 2.0, 06 to 30 Oct 2024) [18]; confirming serotyping (SeqSero 1.2, 13 Jan 2023) [19]; Multilocus Sequence Typing (MLST) (MLST 2.1, 14 Jan 2024) [20, 21]. Regarding ARGs, *S*. Minnesota strains harboring genes from three or more antimicrobial classes were considered multidrug-resistant (MDR) [22].

The five nonclonal *Salmonella* Minnesota isolates from poultry in southeastern Brazil, SPI results were compared by nucleotide alignment with the BLAST Ring Image Generator (BRIG) 0.95 [23], and the Gview server for Blast atlas analysis, conducted on the Gview server [24]; *Salmonella* Typhimurium LT2 (NC_003197.2) was used as a reference strain.

Furthermore, the VFanalyzer was used to identify virulence factor determinants at the Virulence Factors Database (VFDB), accessed on 29 Aug 2024 (*Salmonella* Typhimurium LT2 - chromosome: NC_003197 - was included as reference genome) (Table S2). Additionally, a Venn diagram containing virulence genes of the five nonclonal *S*. Minnesota isolates from poultry in southeastern Brazil was created through the Bioinformatics & Evolutionary Genomics program on 3 Mar 2024.

Further, assembled genomes were analysed by Phage Search Tool with Enhanced Sequence Translation (PHATEST) to identify the presence of prophage sequences [25–27] (accessed on 07 Oct 2024). Only prophages identified as “intact” (score > 90) were considered for the results.

Phylogenetic relationships amongst 268 *S*. Minnesota whole-genome phylogenies were reconstructed using *Salmonella* Typhimurium LT2 (AE006468.2) as outgroup. Further details regarding the metadata of the sequences used in this analysis are available in the Supplementary Table 3 (Table S3). Input genomes were annotated with Prokka1.14.5 [28]. The Roary v 3.12.0 [29] pipeline with default parameters generated by the matrix from the MAFFT alignment program [30], respectively. The phylogenetic tree was constructed using the Maximum Likelihood method in IQ-TREE2 2.2.3 [31] with clade support estimates calculated using ultrafast bootstrap (UFboot) of 1000 pseudo-replicates [32]. GTR + F+I was applied as a best-of-fit model according to the BIC with ModelFinder [33]. The phylogenetic tree was edited in the online tool iTOL—Interactive Tree of Life [34].

### Phenotypic characterization of antimicrobial resistance

The antimicrobial susceptibility test the five nonclonal *S*. Minnesota isolates from poultry in southeastern Brazil, was evaluated by Kirby-Bauer disk diffusion as described by Benevides et al. [35]. *Escherichia coli* strain ATCC^®^ 25,922™ was used as quality control. Fourteen antimicrobials belonging to six different classes were evaluated (Table S4). Clinical and Laboratory Standards Institute reports [36, 37] were used for breakpoints interpretation. According to Firoozeh et al. [38], intermediate profiles were assumed to be resistant.

### Phenotypic evaluation of biofilm-forming ability

The evaluation of the biofilm-forming capacity the five nonclonal *S*. Minnesota isolates from poultry in southeastern Brazil was carried out following the protocol of Vetsby et al. [39]. Each inoculum was prepared separately in Luria-Bertani (LB) agar (Kasvi, Madrid, Spain) and incubated at 37 °C /18 h. After incubation (Luria-Bertani - LB agar, at 37 °C /18 h), the bacterial suspension was adjusted to 0.5 on the McFarland scale on a 0.85% saline solution. Subsequently, the bacterial suspension was diluted 1:10⁵ in saline solution to reach a final concentration of 10³ colony-forming units per milliliter (CFU/mL).

Briefly, into 96-well polystyrene microtiter plates, 100 µL of LB broth supplemented with 10% tryptic soy broth (TSB; Kasvi, Madrid, Spain) and 5% yeast extract (YE) was added, and 30 µL of each inoculum in triplicate was statically incubated at 37 °C for 48 h. Negative (enriched LB broth) and positive controls (*Pseudomonas aeruginosa* ATCC 15442) were included in triplicate.

Planktonic cells were removed by three times washing, and wells were air-dried at room temperature, then stained with 130 µL of 1% crystal violet for 30 min. Excess dye was removed by three additional washes, and plates were dried again at room temperature. Biofilm-associated crystal violet was then solubilized with 130 µL of 30% glacial acetic acid. Absorbance was measured at 495 nm using a microplate reader (IMark, BIO-RAD-Hercules, USA). 

Biofilm formation was quantified by optical density (OD), in triplicate. The cut-off OD (ODc) was defined as three standard deviations above the mean OD of the negative control. Isolates were subsequently classified into four adherence categories, following the criteria of Stepanovic et al. [40]: non-adherent (OD ≤ ODc); weakly adherent (ODc < OD ≤ 2×ODc); moderately adherent (2×ODc < OD ≤ 4×ODc); strongly adherent (OD > 4×ODc).

## Results

### Genome assembly

Quality parameters of the draft genome assemblies from the five nonclonal *S*. Minnesota isolates from poultry in southeastern Brazil are presented in Table 1. Genomes had GC content between 51 and 51.3, and genome sizes from 4,867,437 to 4,935,001 bp. The Seqsero result indicated an antigenic profile currently recognized in the Kauffmann White Scheme 21:b: e,n, x. (compatible with Minnesota serovar), and MLST analysis showed that all genomes belonged to the sequence type 548 (ST548).

**Table 1:** Quality features of draft genome assemblies of the five nonclonal *Salmonella* Minnesota isolates from poultry in southeastern Brazil

_Sample ID_					
_Strain ID_	_**SM_01**_	_**SM_02**_	_**SM_03**_	_**SM_04**_	_**SM_05**_
_Genome/assembly accession numbers_	_SAMN39832342_	_SAMN39832343_	_SAMN39832344_	_SAMN39832345_	_SAMN39832346_
_Genome size (bp)_	_4,935,001_	_4,923,919_	_4,867,437_	_4,908,269_	_4,881,843_
_Contigs number_	_93_	_81_	_66_	_86_	_478_
_% GC_	_51.2_	_51.2_	_51.3_	_51.2_	_51_
_L50_	_12_	_12_	_11_	_16_	_7_
_N50 (bp)_	_156961_	_168434_	_157204_	_110743_	_193736_
_Average depth of coverage_	_48_	_57_	_54_	_57_	_32_
_CDSs_	_4,594_	_4,568_	_4,497_	_4,569_	_4,958_
_rRNA_	_10_	_10_	_10_	_10_	_10_
_tRNA_	_114_	_115_	_114_	_113_	_112_

*N50: represents the sequence length of the shortest contig at which 50% of the total assembly length is reached; L50: represents the minimal count of contigs needed so that their total length equals half of the genome size; CDSs: Coding Sequences; rRNA: Ribosomal Ribonucleic Acid; tRNA: Transfer Ribonucleic Acid

### Antimicrobial resistance determinants

Of the 268 sequences analyzed,191 were considered MDR for carrying three or more resistance genes to antimicrobials from different classes (Table S1). All 268 sequences of *S*. Minnesota harbored the gene *aac(6′)-Iaa* (amikacin and tobramycin), followed by *aph(3’)-Ia* in 36.57% (98/268; lividomycin, ribostamycin, kanamycin, paromomycin, and neomycin) and *aadA1* in 33.58% (90/268; spectinomycin), which determine resistance against the Aminoglycoside antimicrobials’ class.

Other ARGs classes found into *S*. Minnesota genomes from database were: Sulfonamide 69.96% (184/263) with the *sul2* gene in 99.45% (183/184) of these sequences; Tetracycline 69.58% (183/263) with the *tetA* gene in 97.26% (178/183); Beta-lactam 63.49% (167/263) with the *bla*_CMY−2_ in 95.80% (160/167); Quinolone 60.45% (159/263) with all sequences carrying the gene *qnrB19* and 99.23% (261/263) of the sequences in the database harboring a point mutation in ParC [T57S (acc > agc)]. It’s important to highlight that ARG Amphenicol (14/263), Macrolide (13/263), Trimethoprim (12/263), Fosfomycin (4/263), and Polymyxin (3/263) classes were identified in low numbers in the database sequences but not identified in the five nonclonal *S*. Minnesota isolates from poultry in southeastern Brazil.

Regarding beta-lactam antimicrobials, in addition to several *S.* Minnesota sequences carrying the *bla*_CMY−2_ gene, six sequences carried the *bla*_CTX−M−8_ gene, seven carried *bla*_CTX−M−55_ gene, and three presented *bla*_TEM−126_ gene, which are responsible for a large range of beta-lactam drugs, including cefepime, a 4th-generation cephalosporin.

Moreover, three classes of disinfectant resistance genes were identified: *qacE* in 2.28% (6/263) (SM_24, SM_131, SM_132, SM_134, SM_179, and SM_189), *formA* in 1.90% (5/263) (SM_158, SM_159, SM_160, SM_161, and SM_169), and *sitABCD* in 1.14% (3/263) (SM_240, SM_256, and SM_267). These genes encode resistance to quaternary ammonium compounds, aldehydes, and peroxide, respectively.

The five nonclonal *S*. Minnesota isolates from poultry in southeastern Brazil, the ARGs and their antimicrobial classes found, in addition to the aminoglycoside resistance genes described, *bla*_CMY−2,_ which confers resistance to Beta-lactams; *sul2* to Sulfonamide, *qnrB1*, *qnrB19*, and *qnrB81* to Quinolone, and *tetA* to Tetracycline. All five nonclonal *S*. Minnesota isolates from poultry in southeastern Brazil, this study were resistant to Amoxicillin/clavulanic acid, Ampicillin, Ceftazidime, Cefuroxime, and Tetracycline, demonstrating correlation with genotyping results (Table 2). No disinfectant-resistant genes were identified in the nonclonal *S*. Minnesota genomes isolates from poultry in southeastern Brazil.

**Table 2:** Genotypic and phenotypic characterization of antimicrobial resistance profiles of the five nonclonal *Salmonella* Minnesota isolated from poultry in southeastern Brazil

_Sample ID_	_Antimicrobial Resistance phenotypes_	_Antimicrobial Resistance genes_								
		_*aac(6’)−Iaa*_	_*aadA1*_	_*aph(3’)−Ia*_	_*bla*_CMY−2	_*sul2*_	_*tet(A)*_	_*qnrB5*_	_*qnrB19*_	_*qnrB81*_
_SM_01_	_AMC, AMP, CAZ, CRX, CTF, EST, NAL, TET_	_+_	_+_	_+_	_+_	_+_	_+_	_−_	_+_	_−_
_SM_02_	_AMC, AMP, CAZ, CRX, CTF, NAL, TET_	_+_	_−_	_+_	_+_	_+_	_+_	_−_	_+_	_−_
_SM_03_	_AMC, AMP, CAZ, CRX, CTF, EST, TET_	_+_	_+_	_+_	_+_	_+_	_+_	_+_	_+_	_+_
_SM_04_	_AMC, AMP, CAZ, CRX, ENO, NAL, TET_	_+_	_−_	_−_	_+_	_+_	_+_	_−_	_+_	_−_
_SM_05_	_AMC, AMP, CAZ, CRX, EST, TET_	_+_	_+_	_−_	_+_	_+_	_−_	_−_	_+_	_−_

AMC-Amoxicillin/clavulanic acid, AMP-Ampicillin, CAZ-Ceftazidime, CRX-Cefuroxime, CTF-Ceftiofur, ENO-Enrofloxacin, EST-Streptomycin, NAL-Nalidixic acid, TET-Tetracycline

### Pathogenicity islands (SPI) and virulence genes

In total, eight pathogenicity islands and two pathogenic chromosomal regions were identified in the 263 *S.* Minnesota sequences analyzed from the database and the five nonclonal *S*. Minnesota isolates from poultry in southeastern Brazil are described in Table 3.

**Table 3:** *Salmonella* Pathogenicity Islands (SPIs) identified in the 263 *Salmonella* Minnesota sequences analyzed by the SPIfinder, and those identified in the five nonclonal *Salmonella* Minnesota strains isolated from poultry in southeastern Brazil

SPIs - Identified in all 263 Salmonella Minnesota studied genomes										
**Total**	C63PI	Not_named	SPI-1	SPI-2	SPI-3	SPI-4	SPI-5	SPI-9	SPI-13	SPI-14
	263	263	263	263	263	206	263	263	263	263
%	100	100	100	100	100	78.32	100	100	100	100
Strains	**SPIs - Identified in the five nonclonal** ***Salmonella*** **Minnesota isolates from poultry in southeastern Brazil.**									
SM_01	SPI-1, SPI-2, SPI-5, SPI-9, SPI-13, SPI-14									
SM_02	SPI-1, SPI-2, SPI-3, SPI-4, SPI-5, SPI-9, SPI-13, SPI-14									
SM_03	SPI-1, SPI-2, SPI-3, SPI-4, SPI-5, SPI-9, SPI-13, SPI-14									
SM_04	SPI-1, SPI-2, SPI-3, SPI-5, SPI-13, SPI-14									
SM_05	SPI-1, SPI-2, SPI-3, SPI-5, SPI-13, SPI-14									

**Fig. 1:**
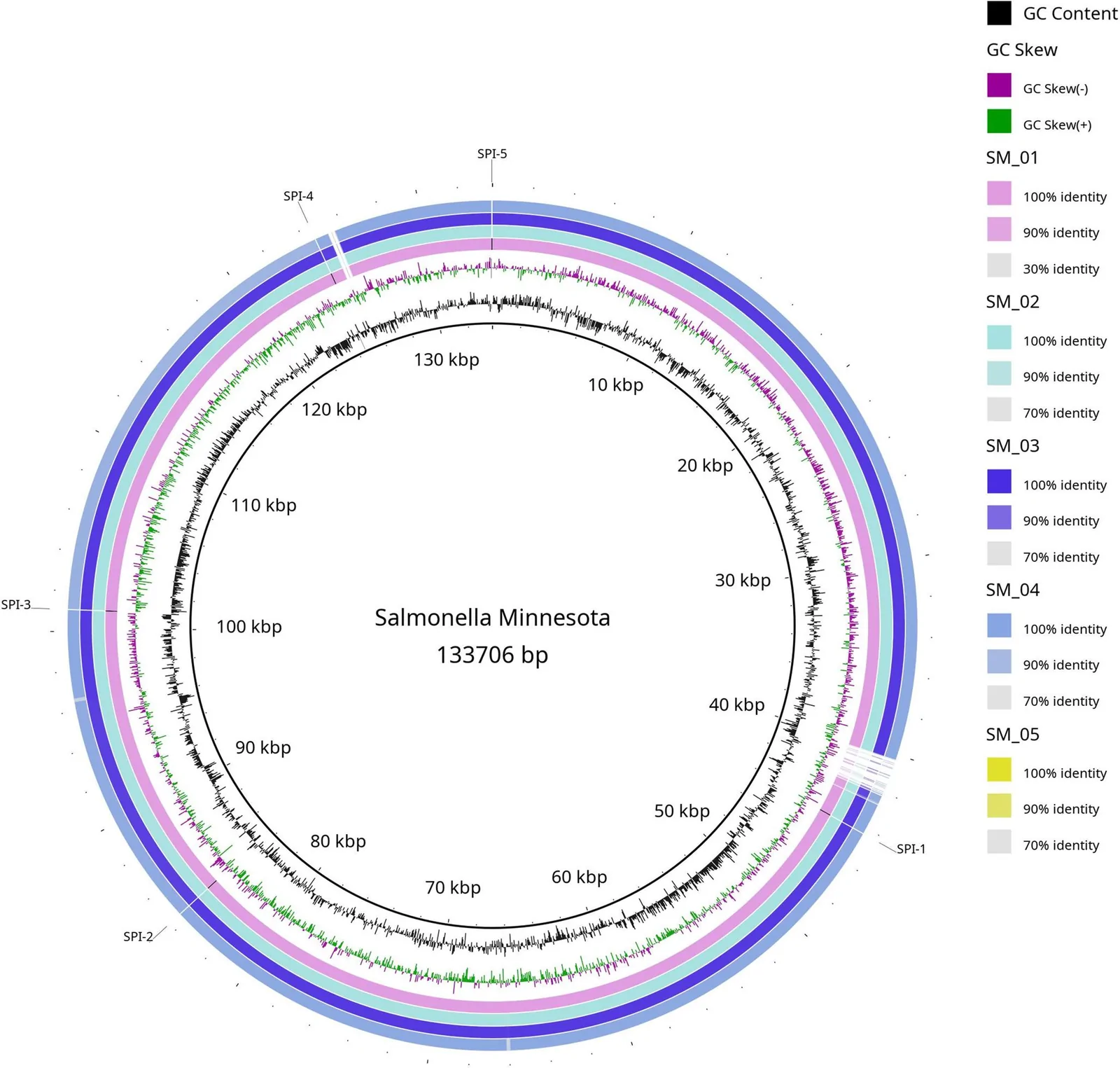
BLAST ring image of the Pathogenicity Islands SPIs 1–5 detected in five nonclonal *Salmonella* Minnesota isolates from poultry in southeastern Brazil. Color intensities represent the percentage of identity (> 90%) with the reference strain *Salmonella* Typhimurium LT2, while blank areas indicate no identity with the reference. The figure is shown in order from inside to outside, starting from the isolates in the right column

**Fig. 2:**
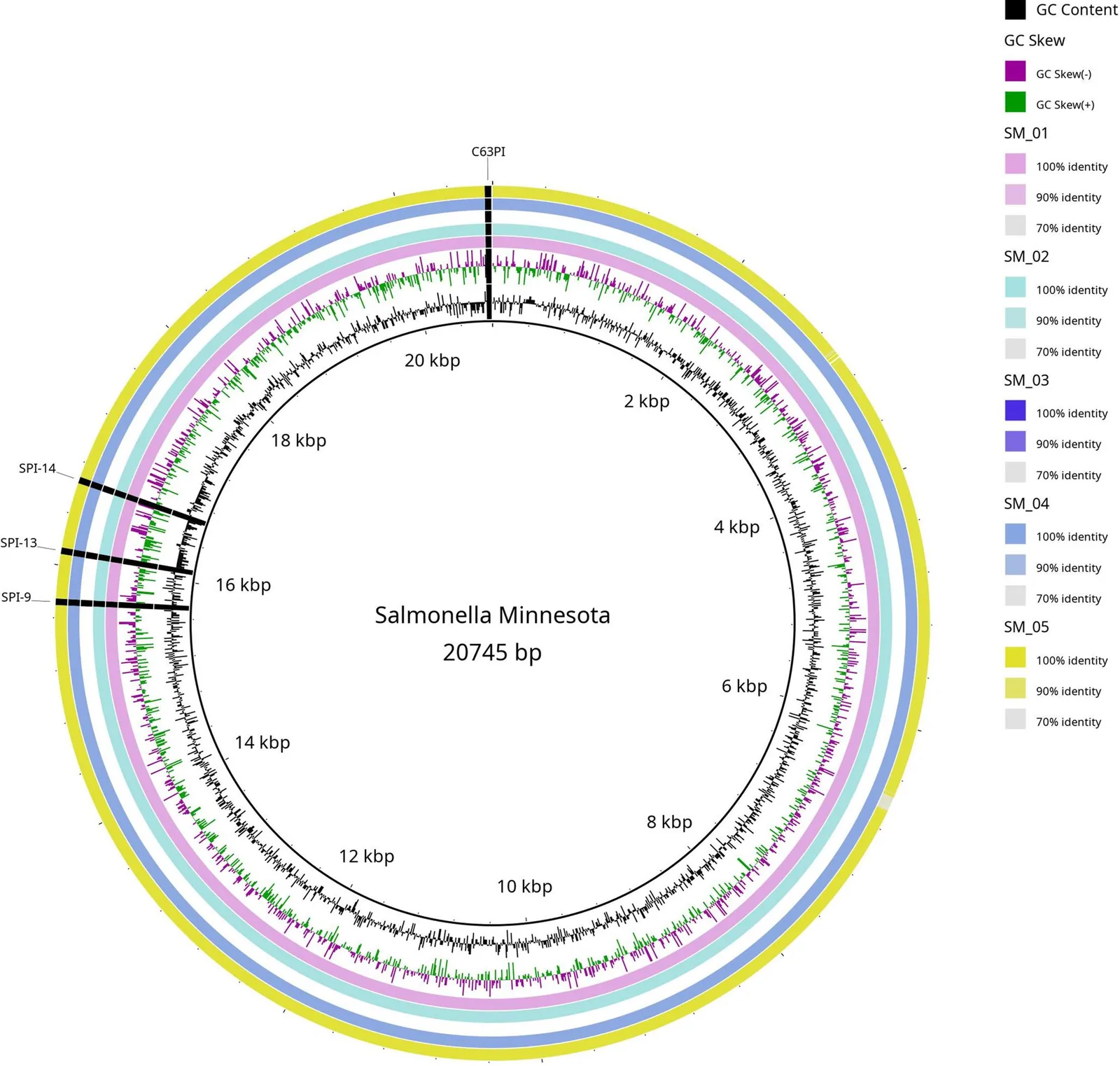
BLAST ring image of the Pathogenicity Islands SPIs SPI-9, 13, 14, and C63PI detected in the five nonclonal *Salmonella* Minnesota isolates from poultry in southeastern Brazil. Color intensities represent the percentage of identity (> 90%) with the reference strain *Salmonella* Typhimurium LT2, while blank areas indicate no identity with the reference. The figure is shown in order from inside to outside, starting from the isolates in the right column

However, a deep investigation on the SPIs 1–5 (Fig. 1) and SPI-9, SPI-13, SPI-14, and C63PI (Fig. 2) revealed an important difference observed in the five nonclonal *S*. Minnesota isolated from poultry in southeastern Brazil: the SPI-4 region is defective in the five sequences on the first base pairs (between 120 and 130 kbp; Fig. 1), and the SPI-5 on the first base pairs as well (between 40 and 50 kbp; Fig. 1). Furthermore, a lack was observed in the SPI-3 (between 90 and 100kbp; Fig. 1) and SPI-1 (between 60 and 70kbp; Fig. 1) regions in the SM_04 sequence. In Fig. 2, only the C63PI genomic region of the SM_05 sequence was observed to have a defect between 6 and 8 kbp.

**Fig. 3:**
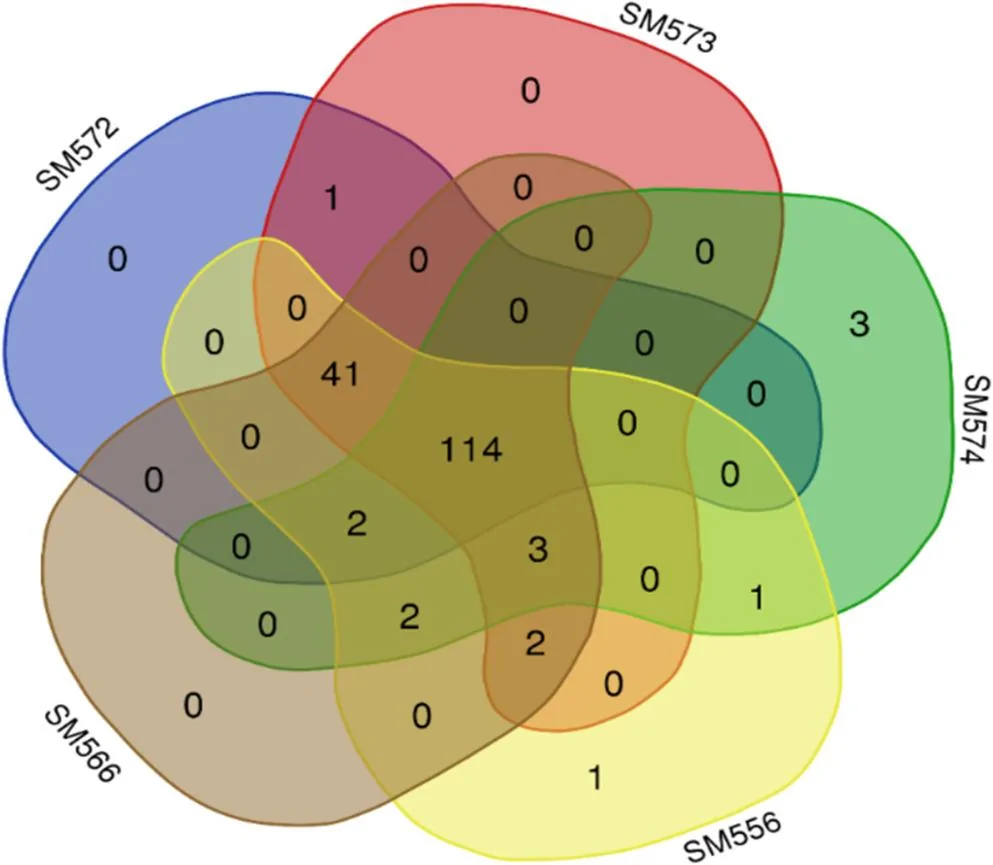
Venn Diagram showing the number of virulence genes shared between the five nonclonal *Salmonella* Minnesota isolates from poultry in southeastern Brazil (http://bioinformatics.psb.ugent.be/webtools/Venn/). SM_01 (SM556), SM_02 (SM566), SM_03 (SM572), SM_04 (SM573) and SM_05 (SM574)

In the comparative analysis of the five genomes of nonclonal *S*. Minnesota from poultry in southeastern Brazil, 114 virulence genes were identified in all five. Another 50 genes are shared between four or three sequences. Moreover, the SM_01 and SM_05 genomes exclusively carry *ssaM* and *safA*, *spiC*/*ssaB*, and *pltB* genes, respectively, while they share the *pegA* gene; SM_03 and SM_04 genomes share the *slrP* gene, as illustrated in Fig. 3 and described in Table 4.

**Table 4:** Virulence genes shared between the five nonclonal *Salmonella* Minnesota isolates from poultry in southeastern Brazil

Samples*	Genes	Total
SM_01, SM_02, SM_03, SM_04 and SM_05	*sipC ssaL stjC sthA sptP spaS faeD csgE ybtQ tcfB fimD safB irp2 sopA sseD sodCL ssaN* * pipB2 invE ssaE ybtS mgtB bcfB invI invG invC sthB sseG hilA csgA ybtX bcfC fimZ sopE2* * ybtT fimH orgB stbD sscA sscB ssaR ssaH sifB iacB ssaP stbB csgG sinH sopD pilW ssaG* * ssaU ssrA sipA pegC ssaK staC stdA tcfD sseJ ssaV sipD tcfC csgB spaP prgJ pegD stjB* * ratB spaR mig-14 PhoQ ssaO invH sseK2 csgD faeH ssrB avrA sseE hilC prgH invA iacP* * sthE sicA faeE pns/fyuA sopD2 sthC staG pegB sseB staB invB sifA sseL staA sprB ssaQ* * lpfC staD invJ hilD sopB/sigD sipB ybtP phoP ssaT stdB galE orgC ybtU*	114
SM_01, SM_02, SM_03 and SM_04	*safC bcfE fimI stbE misL spaQ staF stiC sthD stiH spaO bcfA cdtB stdC faeI mgtC faeC ybtA* * csgF sicP staE pipB safD invF ssaJ sseF fimA bcfG orgA stiB pltA bcfF stbA bcfD stbC prgK* * pefB fimC prgI fimF faeJ*	41
SM_01, SM_02, SM_03 and SM_05	*ssaC ssaD*	2
SM_01, SM_02, SM_04 and SM_05	*csgC fimW ssaS*	3
SM_03 and SM_04	*slrP*	1
SM_01 and SM_05	*pegA*	1
SM_05	*piC/ssaB pltB safA*	3
SM_01	*ssaM*	1
		166

*SM_01 (SM556), SM_02 (SM566), SM_03 (SM572), SM_04 (SM573) and SM_05 (SM574)

Regarding the 263 *S*. Minnesota genomes from the database, 195 genes were identified as belonging to 13 virulence factor (VF) classes (Table 5). These included fimbrial and nonfimbrial adherence determinants, macrophage-inducible genes, magnesium and iron uptake, invasion, regulation, secretion systems, and other determinants. Important to highlight that the *mig-14* gene was identified in all the analyzed sequences, and regulator *phoP* was identified in 73,38% (193/263) of the analyzed sequences. Furthermore, the *cdtB* gene, which encodes the B subunit of the cytolethal distending toxin (CDT), was identified in 99.23% (261/263) of the sequences in the database and four of the *S*. Minnesota isolates from poultry in the Brazilian southeastern region (SM_01, SM_02, SM_03, and SM_04).

**Table 5:** Presence of virulence determinants carried by the 263 *Salmonella* Minnesota sequences through the Virulence Factor Database (VFDB)

Function	Genes
Adherence	*faeC* (68.82%), *faeD* (96.96%), *faeE* (76.81%), *faeE* (98.48%), *faeH* (99.24%), *faeI* (66.92%), *faeJ* (74.14%), *fimB* (2.28%), *galE* (4.56%), *pilQ* (10.65%), *pilR* (10.27%), *pilS* (2.28%), *pilV* (7.98%), *pilW* (60.84%)
Autotransporter	*vat* (0.38%)
Fimbrial adherence determinants	*bcfA* (71.48%), *bcfB* (96.20%), *bcfC* (100%), *bcfD* (100%), *bcfE* (71.48%), *bcfF* (72.24%), *bcfG* (69.58%), *csgA* (72.62%), *csgB* (72.24%), *csgC* (23.57%), *csgD* (73.76%), *csgE* (69.20%), *csgF* (72.62%), *csgG* (100%), *fimA* (74.52%), *fimC* (80.99%), *fimD* (100%), *fimF* (72.62%), *fimH* (100%), *fimI* (71.86%), *fimW* (45.63%), *fimY* (36.12%), *fimZ* (100%), *lpfC* (100%), *pefB* (57.03%), *pefC* (1.14%), *pefD* (0.38%), *pegA* (79.09%), *pegB* (77.19%), *pegC* (100%), *pegD* (100%), *safA* (6.46%), *safB* (99.62%), *safC* (99.62%), *safD* (46.01%), *staA* (74.14%), *staB* (100%), *staC* (100%), *staD* (74.52%), *staE* (72.62%), *staF* (74.14%), *staG* (100%), *stbA* (70.72%), *stbB* (100%), *stbC* (98.86%), *stbD* (98.86%), *stbE* (71.48%), *stdA* (71.86%), *stdB* (99.62%), *stdC* (74.14%), *steA* (5.32%), *sthA* (73%), *sthB* (69.20%), *sthC* (100%), *sthD* (71.10%), *sthE* (100%), *stiA* (72.24%), *stiB* (73%), *stiC* (100%), *stiH* (100%), *stjB* (100%), *stjC* (73%), *tcfA* (30.04%), *tcfB* (73%), *tcfC* (99.62%), *tcfD* (100%)
Invasion	*ibeB* (1.14%)
Iron uptake	*irp2* (66.54%), *iucA* (0.76%), *iucB* (0.76%), *iucC* (1.14%), *iutA* (1.14%), *psn/fyuA* (66.16%), *sitA* (1.14%), *sitB* (1.14%), *sitC* (1.14%), *sitD* (1.14%), *ybtA* (47.15%), *ybtE* (0.76%), *ybtP* (66.54%), *ybtQ* (59.32%), *ybtQ* (7.22%), *ybtS* (0.38%), *ybtS* (65.40%), *ybtT* (66.54%), *ybtU* (66.54%), *ybtX* (65.78%)
Macrophage inducible genes	*mig-14* (100%)
Magnesium uptake	*mgtB* (98.86%), *mgtC* (72.24%)
Nonfimbrial adherence determinants	*misL* (100%), *ratB* (50.95%), *shdA* (65.40%), *sinH* (100%)
Non-LEE encoded TTSS effectors	*espO1-1* (3.04%), *nleC* (0.38%)
Others	*galE* (86.69%)
Regulation	*phoP* (73.38%), *phoQ* (100%)
Secretion system	*avrA* (100%), *hilA* (100%), *hilC* (98.10%), *hilD* (96.58%), *iacP* (77.95%), *iagB* (74.14%), *invA* (100%), *invB* (67.30%), *invC* (100%), *invE* (100%), *invF* (71.86%), *invG* (100%), *invH* (74.52%), *invI* (69.20%), *invJ* (100%), *orgA* (35.74%), *orgB* (75.67%), *orgC* (76.05%), *pipB* (100%), *pipB2* (100%), *prgH* (99.24%), *prgI* (76.43%), *prgJ* (76.43%), *prgK* (73.38%), *sicA* (76.43%), *sicP* (75.29%), *sifA* (100%), *sifB* (99.62%), *sipA* (100%), *sipB* (100%), *sipC* (100%), *sipD* (99.62%), *slrP* (100%), *sopA* (100%), *sopB/sigD* (100%), *sopD* (99.24%), *sopE2* (71.10%), *spaO* (97.34%), *spaP* (78.33%), *spaQ* (73.38%), *spaR* (100%), *spaS* (100%), *spiC/ssaB* (23.57%), *sprB* (72.24%), *sptP* (100%), *ssaC* (100%), *ssaD* (100%), *ssaE* (77.95%), *ssaG* (78.33%), *ssaH* (79.09%), *ssaI* (30.04%), *ssaJ* (77.57%), *ssaK* (74.90%), *ssaL* (100%), *ssaM* (27.76%), *ssaN* (100%), *ssaO* (74,0.2%), *ssaP* (72.24%), *ssaQ* (100%), *ssaR* (68.82%), *ssaS* (20.91%), *ssaT* (58.56%), *ssaU* (100%), *ssaV* (99.62%), *sscA* (67.30%), *sscB* (71.10%), *sseA* (7.98%), *sseB* (78.71%), *sseC* (100%), *sseD* (74.90%), *sseE* (76.43%), *sseF* (100%), *sseG* (100%), *sseJ* (100%), *sseK2* (98.86%), *sseL* (98.86%), *ssrA* (100%), *ssrB* (57.41%)
Toxin	*cdtB* (99.25%), *pltA* (99.24%), *pltB* (6.08%)

Fimbrial and nonfimbrial adherence genes associated with the ability to form biofilms, such as *fimA* (74.52%), *fimH* (100%), *csgA* (72.62%) and *csgD* (73.76%), *pefB* (57.03%), *saf* operon - *safA* (6.46%), *safB* (99.62%), *safC* (99.62%) and *safD* (46.01%) - in addition to *lpfC* (100%), were found in the sequences of *S*. Minnesota from the database (Table 5). Similarly, in the new *S*. Minnesota sequences from this study, the genes *fimH*, *csgA*, *csgD*, *safB*, and *lpfC* were identified in all five sequences; however, the genes *fimA*, *pefB*, *safC*, and *safD* were not identified only in sequence SM_05, and the gene *safA* was identified only in sequences SM_01 and SM_05, as described in Table 4. The complete virulence genes found in *S*. Minnesota genomes are shown in Table S2.

### Biofilm formation capacity of Brazilian *Salmonella* Minnesota

The five nonclonal *S*. Minnesota isolates from poultry in southeastern Brazil were able to form biofilm; however, with different adherence capacities: SM_01 and SM_05 were considered to have weak adherence, while SM_02, SM_03, and SM_04 demonstrated moderate adherence (Table S5).

### Prophage regions and plasmid replicons presence

The five nonclonal *Salmonella* Minnesota isolates from poultry in southeastern Brazil, six different prophage regions were observed, with size (22.2Kb − 270.3Kb) and GC content (49,2–52,88) variable; highlighting that the SM_05 genome did not include the Escherichia_phiV10 (NC_007804) prophage region, which was present in the other four nonclonal *S.* Minnesota isolates from poultry in southeastern Brazil. However, it was the only sequence that included the Shigella_SfIV (NC_022749) in your genome among all genomes evaluated in this study (Table S6 and Table S7). Regarding all 263 analyzed genomes from the database, 53 different prophage regions were identified (Table S7). The phages found embedded in the genomes of *S*. Minnesota have already been described as responsible for disseminating virulence genes, such as those encoding effector proteins involved in the invasion and immune evasion of *Salmonella* serovars [41, 42].

Furthermore, two types of plasmid replicons were found in the five nonclonal *S.* Minnesota isolates from poultry in southeastern Brazil: COL(pHAD28), carrying the *qrnB19* gene, and the plasmid replicon IncC (Table 6). In total, 25 plasmid replicons were identified in the 263 from the Enterobase *S*. Minnesota sequences analyzed, although the most frequent were the same as those exclusive from this study: IncC 69.20% (182/263) and COL(pHAD28) 61,98% (163/263) (Table S2).

### Phylogenetic insights of *Salmonella* Minnesota

**Fig. 4:**
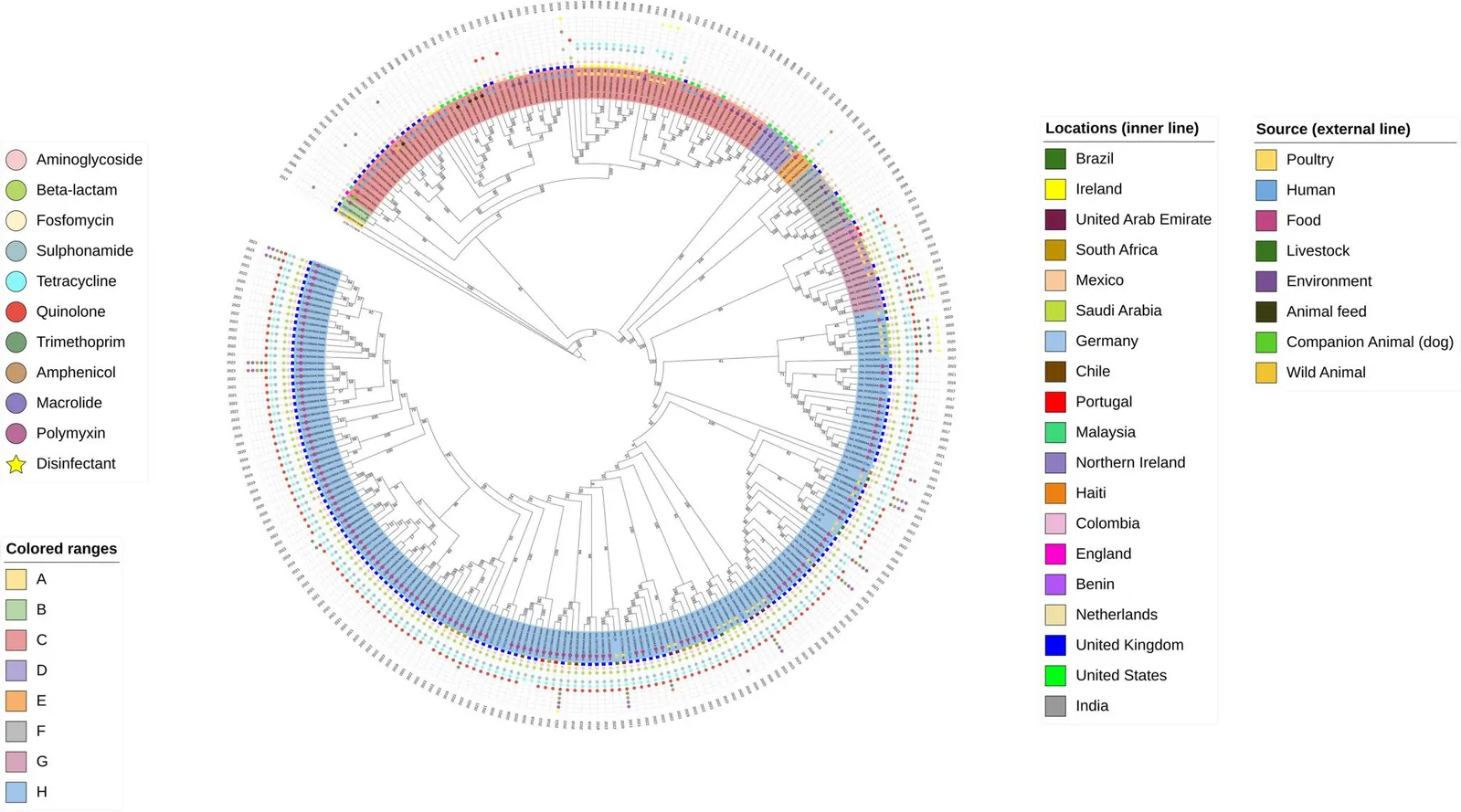
Maximum Likelihood phylogenetic tree of 268 ST548 *Salmonella* Minnesota strains recovered from different sources, countries of origin from the years 2000 to 2024. *Salmonella* Typhimurium LT2 (AE006468.2) was used as the outgroup. The clade support is indicated above or next to each branch as ultrafastbootstrap values, as calculated from 1000 pseudoreplicates. The colors in the inner ring of the tree represent the location of isolation, and the colors in the outer ring indicate the source of isolation (see legend). The colored ranges represent the division of clades: clade **A** (yellow), clade **B** (green), clade **C** (pink), clade **D** (purple), clade **E** (orange), clade **F** (gray), clade **G** (lilac), and clade **H** (blue). The outer balls refer to sequences that carry resistance genes to antimicrobial classes

The pangenome analysis indicated that all *S*. Minnesota ST548 genomes harbored 12,405 genes, comprising 3872 core genes, 183 soft-core genes, 763 shell genes, and 7,587 cloud genes. The Maximum Likelihood tree (Fig. 4) revealed eight clades (A-H), of which two were well-defined clades with the majority of sequences (blue and pink): clade H (blue) comprises 59.69% (157/263) of *S.* Minnesota’s sequences isolated from different sources, countries of origin, and in the period from 2017 to 2024 from database, and the five nonclonal *S.* Minnesota isolates from poultry in southeastern Brazil; clade C (pink) shelter 26.11% (70/268) of the sequences analyzed. The absence of a plateau zone observed in the curves for the pangenome indicates that the core genome set is kept open (Figure S1).

The sequences SM_01 and SM_05 were grouped into a clade with the sequence SAL DD0816 isolated from food in the United Kingdom, and they were with three isolates of human origin (SAL VC8914) and food (SAL VC9641 and SAL VC8906). The SM_02 sequence was grouped into a clade with two food sequences from the United Kingdom (SAL VC9002 and SAL VC8917) and one from poultry in the United Arab Emirates (SAL TC7773). Moreover, the SM_03 sequence was grouped with the SAL RC8774 sequence, isolates from food in the United Kingdom, and five sequences isolates from poultry in Saudi Arabia (SAL WC7020, SAL WC6984, SAL WC6986, SAL WC6988, and SAL WC6987). Finally, the SM_04 sequence claded with three sequenced isolates from poultry from the United Arab Emirates (SAL FD8365 and SAL FD8357) and food from the United Kingdom (SAL GD8613) (Fig. 4).

## Discussion

This study provides the first epidemiological analysis of 268 *S*. Minnesota genomes from diverse sources and their dissemination over time. In the phylogenetic tree, the five nonclonal *S.* Minnesota poultry isolates from southeastern Brazil were closely related to sequences from the United Kingdom and Saudi Arabia (Fig. 4), where outbreaks in humans have been reported [8, 9]. Notably, the first reported human outbreak of *S*. Minnesota occurred in the United Kingdom between 1950 and 1952 and involved 595 clinical cases and two deaths among older adults [8]. These historical findings underscore the capacity of *Salmonella* serovars to cause severe infection, particularly among individuals with compromised immune systems [2–4].

Outbreaks such as those described above can be more difficult to mitigate when caused by multidrug-resistant (MDR) strains. Genes conferring resistance to aminoglycosides (*aac(6’)-Iaa*, *aadA1* and *aph(3’)-Ia*), sulfonamides (*sul2*) and tetracyclines (*tetA*) have been reported in *S*. Minnesota infections [7], representing a major clinical and public health challenge because the development of effective antimicrobial agents has not kept pace with antimicrobial resistance among pathogens [43]. The substantial number of MDR *S.* Minnesota sequences identified in this study is concerning, particularly because some carried genes conferring resistance to next-generation cephalosporins.

The plasmid-mediated *bla*_CMY−2_ and *bla*_CTX−M_ genes identified in several nonclonal *S.* Minnesota sequences from poultry in southeastern Brazil, consistent with previous reports [44, 45], encode CMY and CTX-M enzymes, which are classified as plasmid-mediated AmpC β-lactamases and extended-spectrum β-lactamases, respectively. These enzymes have been reported in human and animal isolates associated with outbreaks worldwide [44, 45]. The ISEcp1 element and Inc plasmids are associated with the dissemination of genes encoding CMY and CTX-M enzymes [45, 46]. Furthermore, the IncC plasmid replicon was the most frequent among the analyzed *S*. Minnesota sequences. In the nonclonal SM_01 poultry isolate from southeastern Brazil, the ISEcp1 element was located within this replicon. Moreover, the same plasmid replicon harbored the conjugative genes *traF* and *traG* in the nonclonal SM_05 genome, also obtained from poultry in southeastern Brazil. These genes encode proteins involved in plasmid transfer from donor to recipient cells through the type IV secretion system [47], thereby facilitating horizontal gene transfer and potentially contributing to the dissemination of antimicrobial resistance [43].

Quinolone resistance is frequently associated with mutations in the *gyrA* and *parC* genes and the expression of *qnr*-type genes encoding plasmid-mediated quinolone resistance [48, 49]. The *qnrB19* gene, carried on the COL(pHAD28) plasmid, was identified in the five nonclonal *S.* Minnesota poultry isolates from southeastern Brazil. Moreover, among EnteroBase sequences containing this plasmid, only four (SM_176, SM_179, SM_209 and SM_257; Table S1) no harbored the *qnrB19* gene. Furthermore, a single point mutation in *ParC* [T57S] was observed in 99.23% (261/263) of the analyzed database sequences and in the five nonclonal *S.* Minnesota poultry isolates from southeastern Brazil, consistent with previous reports [48, 50].

The detection of *Salmonella* Minnesota sequences carrying *qacE*,* formA*, and *sitABCD* genes, which are associated with resistance to disinfectants commonly used in animal production and hospital environments (quaternary ammonium compounds, aldehydes, and peroxide, respectively), represents a potential concern for public and veterinary health [51]. Furthermore, the *sitABCD* operon, located within the C63PI genomic region in database sequences SM_240, SM_256 and SM_267, encodes components of manganese(II) and iron(II) uptake systems that may protect against oxidative stress and detoxify reactive oxygen species (ROS), thereby supporting *Salmonella* survival within immune cells [52].

Many virulence factors in *Salmonella* serovars are encoded within *Salm*on*ella* pathogenicity islands (SPIs), chromosomal regions containing genes involved in invasion, colonization, and immune evasion [48]. SPI-4 and SPI-9 have been implicated in modulating bacterial adhesion to epithelial cells [53, 54]. SPI-4 was not identified in 21.67% (57/263) of the *S.* Minnesota database sequences and was not identified in the poultry isolates nonclonal SM_04 and SM_05 from southeastern Brazil; SPI-9 was also not identified in these two isolates. SPI-4 encodes a type I secretion system (T1SS) responsible for secreting the nonfimbrial adhesin SiiE, which mediates close contact between bacteria and host cells, facilitating the translocation of SPI-1 type III secretion system (T3SS) effector proteins and continued infection [49]. Accordingly, the absence of genes encoded in these regions may attenuate the virulence of these isolates.

The analyzed *S.* Minnesota sequences contained numerous T3SS virulence genes involved in host-cell infection (Table 5); these genes are encoded by SPI-1 and SPI-2, the best-characterized pathogenicity islands [55]. The *ssaM* and *spiC* genes of the SPI-2 T3SS are required for the secretion of translocation proteins SseB and SseD, which form a pore in host membranes [53]. Despite this fundamental role in *Salmonella* infection, the *ssaM* and *spiC* genes were identified in only 27.75% (73/263) and 23.57% (62/263) of the analyzed *S*. Minnesota database sequences, respectively; among the five nonclonal poultry isolates from southeastern Brazil, *ssaM* was detected in SM_01 and *spiC* in SM_05.

The *cdtB* gene, which induces cellular effects in eukaryotic cells, including distension and cell-cycle arrest during human *S*. Typhi infection [56], was identified among 99.23% (261/263) of the analyzed *S*. Minnesota database sequences and four poultry isolates from southeastern Brazil (SM_01, SM_02, SM_03, and SM_04), suggesting that *S*. Minnesota may induce similar cellular lesions. Studies suggest that the *cdtB* gene may have been transferred from *S*. Typhi to nontyphoidal serovars by a prophage that integrates into the bacterial chromosome or through other mechanisms [56, 57]. A wide diversity of prophage homologs from distinct species was identified in the sequences of *S*. Minnesota (Table S6), although their contribution to *Salmonella* virulence remains uncertain.

Biofilm formation may enhance pathogen virulence by promoting infection persistence and protecting pathogens from host immune responses [58, 59]. Biofilms may also facilitate the horizontal transfer of virulence and ARGs between bacterial species [60, 61]. Several biofilm-associated genes (*fimA*,* fimH*,* csgD*,* pefA*,* lpf*) were identified in analyzed the five nonclonal *S.* Minnesota poultry isolates from southeastern Brazil (Table 4) and Enterobase sequences (Table 5). Among the five nonclonal *S.* Minnesota poultry isolates from southeastern Brazil, phenotypic adherence ranged from weak (SM_01 and SM_05) to moderate (SM_02, SM_03 and SM_04) (Table S5). However, further studies are needed to determine which of these genes are expressed.

## Conclusion

This study demonstrated that nonclonal isolates of *S*. Minnesota circulating in poultry in southeastern Brazil harbor genomic features associated with antimicrobial resistance and virulence, supporting the need for public health and veterinary surveillance of this serovar. In addition, the Brazilian isolates were genetically related to isolates from diverse sources worldwide, including isolates associated with human outbreaks. However, further studies are needed to determine whether the acquisition of additional genes contributes to severe human infection or host adaptation, once analyses of the core and accessory genomes suggest that the pangenome of this serovar remains open.

## Supplementary Information

Below is the link to the electronic supplementary material.

Supplementary Material 1. Table S1: Metadata for the 263 *Salmonella* Minnesota genomes retrieved from the Enterobase database and the five nonclonal *Salmonella* Minnesota isolates from poultry in southeast Brazil to perform genomic analysis

Supplementary Material 2. Table S2: The presence (1) or absence (0) of virulence genes among all *Salmonella* Minnesota isolates. The strains are clustered by their prediction of gene similarities. *Salmonella* Typhimurium LT2 (chromosome: NC_003197) was used as the reference genome

Supplementary Material 3. Table S3: Metadata for the 268 *Salmonella* Minnesota genomes phylogenetic analysis (NCBI accession number, year, source, country, and sequence coverage)

Supplementary Material 4. Table S4: Antimicrobial drugs and their class used for susceptibility tests on the five *Salmonella* Minnesota isolates from poultry in the Brazilian southeastern region

Supplementary Material 5. Table S5: Optical density (OD) measured from biofilm formation on polystyrene microtiter plates of the five *Salmonella* Minnesota isolates from poultry in the Brazilian southeastern region

Supplementary Material 6. Table S6: Genomic features of prophages detected in the five *Salmonella* Minnesota isolates from poultry in the Brazilian southeastern region

Supplementary Material 7. Table S7: Genomic features of prophages detected in 263 *Salmonella* Minnesota genomes were analyzed from Enterobase

Supplementary Material 8. Figure S1: Pangenome analysis of 268 *Salmonella* Minnesota, including the new five sequences from poultry in southeastern Brazil

## Data Availability

The assembled genomes of five *Salmonella* Minnesota isolates were deposited at the NCBI Sequence Read Archive (SRA) website, under Project PRJNA1015686. The complete genome data of SM_01, SM_02, SM_03, SM_04, and SM_05 were deposited in GenBank with Accession No. SAMN39832342, SAMN39832343, SAMN39832344, SAMN39832345 and SAMN39832346, respectively.
