# Supplementary material for: Genotypic and phylogenetic characterization of Salmonella Minnesota ST548 strains with potential risk to public health
Source: Braz J Microbiol. 2026 Oct 23;57(1):303. doi: 10.1007/s42770-026-02071-y (PMC13694517; doi:10.1007/s42770-026-02071-y)
Supplement: Supplementary file 1 [file 42770_2026_2071_MOESM4_ESM.docx]

Table S4: Antimicrobial drugs and their class used for susceptibility tests used the five *Salmonella* Minnesota isolates from poultry in southeastern of Brazil.

| Class | Antimicrobial | Concentration (µg/mL) | Manufacturer^1^ |
| --- | --- | --- | --- |
| Quinolones and Fluoroquinolones | Nalidixic Acid | 30 | Sensifar^®^ |
|  | Ciprofloxacin | 5 | Oxoid^®^ |
|  | Enrofloxacin | 5 | Sensifar^®^ |
|  | Ofloxacin | 5 | Sensifar^®^ |
| Phenicols | Chloramphenicol | 30 | Sensifar^®^ |
| Aminoglycosides | Streptomycin | 10 | Sensibiodisc^®^ |
|  | Gentamicin | 10 | Oxoid^®^ |
| Sulfonamides | Trimethoprim/Sulfamethoxazole | 25 | Oxoid^®^ |
| β-lactams | Ceftiofur | 30 | Sensifar^®^ |
|  | Amoxicillin/Clavulanic Acid | 30 | Oxoid^®^ |
|  | Ampicillin | 10 | Sensibiodisc^®^ |
|  | Ceftazidime | 30 | Sensifar^®^ |
|  | Cefuroxime | 30 | Sensifar^®^ |
| Tetracyclines | Tetracycline | 30 | Sensifar^®^ |

^1^Oxoid^®^, Basingstoke, Hampshire, UK; Sigma-Aldrich^®^, St. Louis, Missouri, USA; Sensi-disc^®^, Franklin Lakes, Nova Jersey, EUA; Sensibiodisc^®^, São Paulo, BR; Sensifar^®^, São Paulo, BR
