# Supplementary material for: Genotypic and phylogenetic characterization of Salmonella Minnesota ST548 strains with potential risk to public health
Source: Braz J Microbiol. 2026 Oct 23;57(1):303. doi: 10.1007/s42770-026-02071-y (PMC13694517; doi:10.1007/s42770-026-02071-y)
Supplement: Supplementary file 2 [file 42770_2026_2071_MOESM5_ESM.docx]

**Table S5**. Optical density (OD) measured from biofilm formation on polystyrene microtiter plates of the five *Salmonella* Minnesota isolates from poultry in southeast Brazil,

| **Sample** | **NC** | **SM_01** | **SM_02** | **SM_03** | **SM_04** | **SM_05** | **PA** |
| --- | --- | --- | --- | --- | --- | --- | --- |
| **OD 1** | 0.145 | 0.279 | 0.418 | 0.369 | 0.329 | 0.307 | 3.248 |
| **OD 2** | 0.153 | 0.294 | 0.444 | 0.460 | 0.399 | 0.293 | 2.233 |
| **OD 3** | 0.156 | 0.265 | 0.297 | 0.412 | 0.353 | 0.290 | 2.583 |
| **Mean** | 0.151 | 0.279 | 0.386 | 0.413 | 0.360 | 0.296 | 2.688 |

(NC) Negative control; (PA - Positive Control) *Pseudomonas aeruginosa*; ODc = 0.151.
