# Supplementary material for: Genotypic and phylogenetic characterization of Salmonella Minnesota ST548 strains with potential risk to public health
Source: Braz J Microbiol. 2026 Oct 23;57(1):303. doi: 10.1007/s42770-026-02071-y (PMC13694517; doi:10.1007/s42770-026-02071-y)
Supplement: Supplementary file 3 [file 42770_2026_2071_MOESM6_ESM.docx]

**Table S6.** Genomic features of prophages detected in the five *Salmonella* Minnesota isolates from poultry in southeast Brazil.

| **ID sample** | **Phage identity** | **Access number** | **Contig** | **Region Position** | **Region Length** | **% GC** |
| --- | --- | --- | --- | --- | --- | --- |
| SM_01 | Escherichia_phiV10 | NC_007804 | 2 | 175,284 – 431,437 | 256,1Kb | 51.79 |
|  | Shigella_SfII | NC_021857 | 19 | 2,366,968 – 2,562,801 | 195,8Kb | 51.41 |
|  | Escherichia_500465_1 | NC_049342 | 35 | 3,779,533 – 3,801,791 | 22,2Kb | 51.41 |
| SM_02 | Salmonella_Fels_1 | NC_010391 | 2 | 304,356 – 572,732 | 268,3Kb | 49.39 |
|  | Escherichia_phiV10 | NC_007804 | 18 | 2,718,516 – 2,919,972 | 201,4Kb | 51.77 |
|  | Escherichia_500465_1 | NC_049342 | 40 | 4,469,171 – 4,491,430 | 22,2Kb | 51.41 |
|  | Klebsiella_4LV2017 | NC_047818 | 51 | 4,738,396 – 4,761,143 | 22,7Kb | 52.88 |
| SM_03 | Escherichia_phiV10 | NC_007804 | 12 | 1556846 – 1,827,148 | 270,3Kb | 51.77 |
|  | Escherichia_500465_1 | NC_049342 | 44 | 4,675,139 – 4,699,460 | 24,3Kb | 51.41 |
| SM_04 | Shigella_SfII | NC_021857 | 4 | 798,330 – 993,887 | 195,5Kb | 51.63 |
|  | Escherichia_phiV10 | NC_007804 | 29 | 3,355,758 – 3,481,755 | 125,9Kb | 51.16 |
|  | Salmonella_Fels_1 | NC_010391 | 43 | 4,452,098 – 4,604,720 | 152,6Kb | 49.2 |
| SM_05 | Shigella_SfIV | NC_022749 | 19 | 511,500 – 545,701 | 34,2Kb | 50.71 |
