# Supplementary material for: Genotypic and phylogenetic characterization of Salmonella Minnesota ST548 strains with potential risk to public health
Source: Braz J Microbiol. 2026 Oct 23;57(1):303. doi: 10.1007/s42770-026-02071-y (PMC13694517; doi:10.1007/s42770-026-02071-y)
Supplement: Supplementary file 4 [file 42770_2026_2071_MOESM7_ESM.docx]

**Table S7.** Genomic features of prophages detected in 263 *Salmonella* Minnesota genomes were analyzed from Enterobase.

| **most_common_phage** | **Phage** | **GenBank accession number** | **Count** | **Percentage** |
| --- | --- | --- | --- | --- |
| PHAGE_Burkho_BcepMu_NC_005882 | Burkholderia_BcepMu | NC_005882 | 2 | 0,76 |
| PHAGE_Burkho_phiE255_NC_009237 | Burkholderia_phiE255 | NC_009237 | 1 | 0,38 |
| PHAGE_Cronob_ENT47670_NC_019927 | Cronobacter_ENT47670 | NC_019927 | 68 | 25,86 |
| PHAGE_Cronob_ESSI_2_NC_047854 | Cronobacter_ESSI_2 | NC_047854 | 1 | 0,38 |
| PHAGE_Entero_186_NC_001317 | Enterobacteria_186 | NC_001317 | 1 | 0,38 |
| PHAGE_Entero_fiAA91_ss_NC_022750 | Enterobacteria_fiAA91 | NC_022750 | 7 | 2,66 |
| PHAGE_Entero_I2_2_NC_001332 | Enterobacteria_I2_2 | NC_001332 | 1 | 0,38 |
| PHAGE_Entero_Mu_NC_000929 | Enterobacteria_Mu | NC_000929 | 3 | 1,14 |
| PHAGE_Entero_P88_NC_026014 | Enterobacteria_P88 | NC_026014 | 5 | 1,90 |
| PHAGE_Entero_phi80_NC_021190 | Enterobacteria_phi80 | NC_021190 | 1 | 0,38 |
| PHAGE_Entero_phiT5282H_NC_049429 | Enterobacteria_phiT5282H | NC_049429 | 1 | 0,38 |
| PHAGE_Entero_Sf101_NC_027398 | Enterobacteria_Sf101 | NC_027398 | 1 | 0,38 |
| PHAGE_Entero_SfI_NC_027339 | Enterobacteria_SfI | NC_027339 | 1 | 0,38 |
| PHAGE_Entero_SfMu_NC_027382 | Enterobacteria_SfMu | NC_027382 | 1 | 0,38 |
| PHAGE_Entero_SfV_NC_003444 | Enterobacteria_SfV | NC_003444 | 19 | 7,22 |
| PHAGE_Entero_ST104_NC_005841 | Enterobacteria_ST104 | NC_005841 | 1 | 0,38 |
| PHAGE_Entero_Tyrion_NC_031077 | Enterobacteria_Tyrion | NC_031077 | 5 | 1,90 |
| PHAGE_Entero_UAB_Phi20_NC_031019 | Enterobacteria_UAB_Phi20 | NC_031019 | 34 | 12,93 |
| PHAGE_Erwini_vB_EhrS_59_NC_048198 | Erwinia_vB_EhrS_59 | NC_048198 | 14 | 5,32 |
| PHAGE_Escher_500465_1_NC_049342 | Escherichia_500465_1 | NC_049342 | 121 | 46,01 |
| PHAGE_Escher_D108_NC_013594 | Escherichia_D108 | NC_013594 | 3 | 1,14 |
| PHAGE_Escher_phiV10_NC_007804 | Escherichia_phiV10 | NC_007804 | 157 | 59,70 |
| PHAGE_Escher_pro483_NC_028943 | Escherichia_pro483 | NC_028943 | 1 | 0,38 |
| PHAGE_Escher_RCS47_NC_042128 | Escherichia_RCS47 | NC_042128 | 5 | 1,90 |
| PHAGE_Escher_SH2026Stx1_NC_049919 | Escherichia_SH2026Stx1 | NC_049919 | 1 | 0,38 |
| PHAGE_Phage_Gifsy_1_NC_010392 | Gifsy_1 | NC_010392 | 5 | 1,90 |
| PHAGE_Phage_Gifsy_2_NC_010393 | Gifsy_2 | NC_010393 | 8 | 3,04 |
| PHAGE_Haemop_SuMu_NC_019455 | Haemophilus_SuMu | NC_019455 | 8 | 3,04 |
| PHAGE_Klebsi_4LV2017_NC_047818 | Klebsiella_4LV2017 | NC_047818 | 29 | 11,03 |
| PHAGE_Klebsi_phiKO2_NC_005857 | Klebsiella_phiKO2 | NC_005857 | 1 | 0,38 |
| PHAGE_Klebsi_ST13_OXA48phi12.1_NC_049453 | Klebsiella_ST13_OXA48phi12.1 | NC_049453 | 1 | 0,38 |
| PHAGE_Mannhe_vB_MhM_3927AP2_NC_028766 | Mannheimia_vB_MhM_3927AP2 | NC_028766 | 3 | 1,14 |
| PHAGE_Salmon_118970_sal3_NC_031940 | Salmonella_118970_sal3 | NC_031940 | 9 | 3,42 |
| PHAGE_Salmon_118970_sal4_NC_030919 | Salmonella_118970_sal4 | NC_030919 | 23 | 8,75 |
| PHAGE_Salmon_BTP1_NC_042346 | Salmonella_BTP1 | NC_042346 | 4 | 1,52 |
| PHAGE_Salmon_Fels_1_NC_010391 | Salmonella_Fels_1 | NC_010391 | 113 | 42,97 |
| PHAGE_Salmon_Fels_2_NC_010463 | Salmonella_Fels_2 | NC_010463 | 10 | 3,80 |
| PHAGE_Salmon_SEN1_NC_029003 | Salmonella_SEN1 | NC_029003 | 1 | 0,38 |
| PHAGE_Salmon_SEN34_NC_028699 | Salmonella_SEN34 | NC_028699 | 8 | 3,04 |
| PHAGE_Salmon_SEN8_NC_047753 | Salmonella_SEN8 | NC_047753 | 37 | 14,07 |
| PHAGE_Salmon_SI7_NC_049460 | Salmonella_SI7 | NC_049460 | 8 | 3,04 |
| PHAGE_Salmon_SJ46_NC_031129 | Salmonella_SJ46 | NC_031129 | 2 | 0,76 |
| PHAGE_Salmon_SP_004_NC_021774 | Salmonella_SP_004 | NC_021774 | 3 | 1,14 |
| PHAGE_Salmon_SPN3UB_NC_019545 | Salmonella_SPN3UB | NC_019545 | 9 | 3,42 |
| PHAGE_Salmon_ST64T_NC_004348 | Salmonella_ST64T | NC_004348 | 3 | 1,14 |
| PHAGE_Salmon_SW9_NC_049459 | Salmonella_SW9 | NC_049459 | 2 | 0,76 |
| PHAGE_Salmon_UPF_BP1_NC_047875 | Salmonella_UPF_BP1 | NC_047875 | 2 | 0,76 |
| PHAGE_Salmon_vB_SemP_Emek_NC_018275 | Salmonella_vB_SemP_Emek | NC_018275 | 10 | 3,80 |
| PHAGE_Salmon_vB_SosS_Oslo_NC_018279 | Salmonella_vB_SosS_Oslo | NC_018279 | 19 | 7,22 |
| PHAGE_Stx2_c_1717_NC_011357 | Shiga_Toxin2_c_1717 | NC_011357 | 1 | 0,38 |
| PHAGE_Shigel_SfII_NC_021857 | Shigella_SfII | NC_021857 | 58 | 22,05 |
| PHAGE_Vibrio_VBM1_NC_020850 | Vibrio_VBM1 | NC_020850 | 1 | 0,38 |
| PHAGE_Vibrio_X29_NC_024369 | Vibrio_X29 | NC_024369 | 11 | 4,18 |
